# Survival Assessment by Central Review vs. Local Investigator in Metastatic Melanoma: A Systematic Review and Meta-Analysis †

**DOI:** 10.3390/cancers18040710

**Published:** 2026-02-22

**Authors:** Islam Eljilany, Eissa Jafari, Abdullah Alhumaid, Zeynep Eroglu, Andrew S. Brohl, Lilit Karapetyan, Joseph Markowitz, Nikhil I. Khushalani, Patrick Hwu, Ahmad A. Tarhini

**Affiliations:** 1 Department of Cutaneous Oncology, H. Lee Moffitt Cancer Center and Research Institute, Tampa, FL 33612, USA; 2 Department of Pharmacy Practice, College of Pharmacy, Jazan University, Jazan 45142, Saudi Arabia; 3 Department of Pharmacotherapy and Translational Research, Center for Pharmacogenomics and Precision Medicine, College of Pharmacy, University of Florida, Gainesville, FL 32610, USA; 4 Department of Clinical Pharmacy, College of Pharmacy, University of Hail, Hail 81422, Saudi Arabia; 5 Department of Immunology, H. Lee Moffitt Cancer Center and Research Institute, Tampa, FL 33612, USA

**Keywords:** blinded independent central review, local evaluation, melanoma, meta-analysis, progression-free survival, randomized clinical trial, systematic review, tumor response assessment

## Abstract

**Background:** Although blinded independent central review (BICR) can reduce assessment variability, it introduces additional financial and logistical burdens to trial operations. This study analyzed the discrepancy indexes (DIs) to evaluate differences between progression-free survival (PFS) assessments by local investigators (LIs) and BICR in randomized clinical trials (RCTs) of patients with metastatic melanoma. **Methods:** A comprehensive literature search was conducted on PubMed, Embase, and Cochrane databases up to 30 June 2024. The primary outcome was the DI, which was calculated for each trial as a ratio of the hazard ratios (HR)_BICR_ by HR_LI_. The agreement between PFS HRs was also evaluated using the intraclass correlation coefficient (ICC) and Pearson’s correlation coefficient (*r*). **Results:** Twelve studies comprising 4915 patients were included in this study. Of these, 10 (83%) were Phase III, 11 (92%) were cutaneous melanoma, one was uveal, and all identified PFS as the primary endpoint. Most (86%) of the PFS comparisons yielded the same statistical inference by both BICR and LIs. The overall combined DI was calculated at 1.08 (95% CI: 1.01–1.15), indicating a statistically significant, numerically small difference in PFS evaluations driven primarily by the uveal Phase III double-blinded study, while there was a strong overall correlation [(ICC: 0.87, *p* < 0.001); (*r* = 0.89, 95% CI 0.67–0.96, *p* < 0.0001)]. Cutaneous melanoma trials demonstrated strong agreement between BICR and local investigator assessments. **Conclusions:** In randomized trials of metastatic cutaneous melanoma, LI-assessed PFS closely aligns with BICR and provides equivalent trial-level conclusions in most cases. These findings support the use of LI-assessed PFS as a valid and practical primary endpoint, without routine requirement for BICR. Central review should be reserved for selected scenarios.

## Simple Summary

There is a need to investigate the optimal endpoints and clinical trial designs that may be best suited to accelerate progress in immunooncology drug development. In this study, we were interested in assessing the endpoint of progression-free survival (PFS) as assessed by blinded independent central review (BICR), and of that assessed by local investigators (LI) in randomized clinical trials (RCTs). This study was developed to analyze the discrepancy indexes (DIs) to evaluate differences between PFS assessments by LIs and BICR in RCTs of patients with metastatic melanoma. This systematic review and meta-analysis of 12 RCTs (11 in cutaneous melanoma and one uveal) included 4915 participants. In this systematic review and meta-analysis of 12 randomized trials including 4915 patients, we found strong agreement between LI- and BICR-assessed PFS, particularly in cutaneous melanoma. Differences between the two assessment methods were small and rarely changed trial conclusions. These findings support the use of LI-assessed PFS as a reliable primary endpoint in most cutaneous melanoma trials, with BICR reserved for selected situations where assessment uncertainty is higher.

## 1. Introduction

Progression-free survival (PFS), along with other survival endpoints, including recurrence-free survival (RFS), distant metastasis-free survival, and event-free survival (EFS), has been extensively implemented as a surrogate endpoint for overall survival (OS) in clinical trials in oncology [1,2]. Notably, PFS is measured by calculating the time between randomization and the first signs of disease progression or death, whichever occurs first [3]. As per the Response Evaluation Criteria in Solid Tumors (RECIST) guideline [4], early signs of progression might be explained as a 20% increase in the target lesion diameter or a significant increase in non-target lesions [3]. On the other hand, utilizing OS as an endpoint comes with some challenges, such as the need for more extended follow-up periods and a larger sample size, which might not be feasible [5]. Moreover, the emergence of novel targeted therapies in cancer, where trials of those agents often aim to halt disease progression as a primary endpoint instead of OS due to confounding factors in the post-trial salvage setting, drives the recent increase in the adoption of PFS as a primary clinical trial endpoint [6]. However, a thoughtful implementation of PFS as a primary endpoint in clinical trials is essential in order to avoid certain biases in disease response assessment, PFS evaluation, and operator bias [7,8]. Such factors that may lead to bias tend to result in a higher variability rate in assessing PFS, and may lead to inaccurate clinical conclusions [9].

In the assessment of PFS, investigators implement two well-established tumor assessment strategies in clinical trials: blinded independent central review (BICR) and the local investigators (LIs) assessment. Although BICR is the most widely used method in trials, its superiority over LIs is debatable [10,11]. Previous systematic reviews and meta-analyses compared BICR versus LIs in PFS assessment trials of various solid tumors and showed no significant differences between the two strategies [12,13,14]. However, these studies were conducted in populations with a broad range of tumor types, where differences between BICR and LIs in a specific tumor could be overlooked. Recently, a meta-analysis was conducted by Jacobs F et al. [15] that compared PFS assessment by BICR versus LIs in metastatic breast cancer (mBC) trials and concluded that LIs were more reliable than BICR in assessing PFS in this population. This outcome might prompt investigators to consider comparing BICR and LIs in a more specific patient population with a specific tumor type. Therefore, we conducted this systematic review and meta-analysis to investigate the differences in PFS assessment between BICR and LIs in randomized clinical trials in patients with metastatic melanoma.

## 2. Materials and Methods

This systematic review and meta-analysis was registered in the PROSPERO database (ID: CRD42024578275) and conducted following the PRISMA guidelines (Table S1 in the Supplementary Materials) [16]. No deviations from the pre-registered PROSPERO protocol were made.

### 2.1. Study Objectives

This systematic review and meta-analysis aimed to compare the discrepancy index (DI) of PFS as assessed by BICR versus LIs across all randomized clinical trials published up to 2024 involving metastatic melanoma patients, regardless of treatment type.

### 2.2. Search Strategy and Data Extraction

A comprehensive search was carried out in the PubMed (RRID: SCR_004846), Embase (RRID: SCR_001650), and Cochrane databases (RRID: SCR_013000) up to 30 June 2024, to identify eligible records. The search covered articles utilizing the patient, intervention, comparator, and outcome (PICO) framework [17]. Search strategies were developed based on specified rules and vocabulary for each database (Tables S1–S4 in the Supplementary Materials). The literature was screened blindly and independently by two investigators (E.J., A.A.), and in cases of disagreement, a consensus was obtained from a third investigator (I.E.).

### 2.3. Study Eligibility Criteria

Studies were included in the meta-analysis if they met these criteria: (1) Phase II or III randomized clinical trial (RCT) with published and available data in any of the above databases; (2) studies that included patients with metastatic melanoma; (3) studies with available information on PFS either as a primary or secondary endpoint, assessed by LIs and by BICR; (4) studies published in the English language. Phase I trials were excluded due to dose-escalation designs.

### 2.4. Data Extraction

For each of the eligible studies, the following variables were extracted by three investigators (I.E., E.J., A.A.): first author, year of publication, trial start year, masking design, randomization ratio, trial phase, melanoma type, country/region, sponsor, RECIST criteria version used, sample size (number of patients in the entire population), treatment name in the experimental and control arms, PFS endpoint (primary or secondary), PFS follow-up time, PFS hazard ratios (HRs) and 95% confidence intervals (CIs) assessed by LIs and BICR, and LIs and BICR significance level. In the case of multiple publications for the same trial, the one with the longest follow-up period or the most recent data was included.

### 2.5. Risk of Bias Assessment

The risk of bias in each included study was evaluated by two independent investigators (I.E., E.J.) using the Cochrane Risk of Bias tool v.2 (RoB 2) available at https://www.riskofbias.info/welcome/rob-2-0-tool/current-version-of-rob-2 (accessed on 5 September 2024) [18]. The evaluation covered five domains: randomization process, deviations from intended interventions, missing outcome data, outcome measurement, and selection of reported results. Based on these domains, each study was classified as having a low, moderate, or high risk of bias.

### 2.6. Statistical Analysis

Clinical trial characteristics were reported using descriptive statistics. Categorical variables were presented as frequencies and percentages, with comparisons using the Chi-square test. Continuous variables were presented as medians and inter-quartile ranges, with comparisons between groups using the Mann–Whitney or Kruskal–Wallis test as appropriate. The magnitude of agreement of the estimated effect of PFS by BICR and the LIs for each trial was assessed using the DI. The DI was calculated as the ratio of the BICR-assessed HR to the LI-assessed HR. To assess the variability between the HR_BICR_ and HR_LI_ PFS, a log-transformed HR (logHR) of PFS from LIs and BICR was calculated. Then Pearson’s correlation coefficient (*r*) was performed using logHR_BICR_ as the dependent variable and logHR_LI_ as the independent variable, with the coefficient of determination indicating the proportion of variability in logHR_BICR_ explained by logHR_LI_. The intraclass correlation coefficient (ICC) was calculated to assess the level of agreement between the HR from LIs and BICR using a mixed-effects model. Cochran’s Q test was performed to test for heterogeneity across studies, and *I^2^* was calculated to quantify the degree of heterogeneity. Based on Cochran’s Q test results, a random-effects meta-analysis was performed if significant heterogeneity was detected (*p*-value < 0.05); otherwise, a fixed-effects model was used. For the subgroup analysis, a meta-regression was conducted using stepwise linear regression to determine the most significant variable to be adjusted in the meta-regression model. A funnel plot was used to evaluate the potential publication bias in the included studies. All data preparation, variable creation, prediction models, and figures were created using R version 4.3.3 (R Foundation for Statistical Computing) and SAS version 9.4 (RRID: SCR_008567) (SAS Institute Inc., Cary, NC, USA).

### 2.7. Data Availability

The data used to generate this study’s findings are publicly available, and the codes used to analyze them are available upon request from the corresponding author.

### 2.8. Discrepancy Index

The DI, defined as the ratio of the hazard ratio estimated by BICR to that estimated by LIs, was used in this study as a descriptive measure of concordance between two assessment approaches rather than as a direct measure of bias. A DI value different from 1.0 indicates numerical divergence between effect estimates, but does not, by itself, imply systematic bias or intentional over- or under-estimation by either assessment method. Differences in censoring algorithms, confirmation requirements for progression, imaging assessment schedules, and adjudication procedures between BICR and local investigator evaluations can mechanically result in DI values different from 1, even in the absence of systematic assessment bias.

## 3. Results

### 3.1. Study Characteristics

Out of the 2209 records initially retrieved from the databases, a total of 12 studies [19,20,21,22,23,24,25,26,27,28,29,30] involving 4915 patients were included in this meta-analysis (Figure S1 in the Supplementary Materials). The detailed characteristics of these studies are summarized in Table 1. The studies span from 2014 to 2023, with the majority [19,20,21,22,23,24,25,26,27,28] being Phase III trials (n = 10, 83%) and most [19,21,22,23,24,26,30] employing an open-label design (n = 7, 58%). For PFS evaluation, RECIST version 1.1 was used in 92% of the studies (n = 11) [19,20,21,22,23,24,25,27,28,29,30]. Importantly, PFS was the primary endpoint across all studies, with follow-up periods ranging from 8 to 60 months post-randomization. The majority of studies [19,21,22,23,24,25,26,27,28,29,30] (n = 11, 92%) focused on patients with cutaneous melanoma, while one study [20] included participants with uveal melanoma.

### 3.2. Discrepancy Index Between PFS Assessed by LIs and by BICR

**Figure 1:**
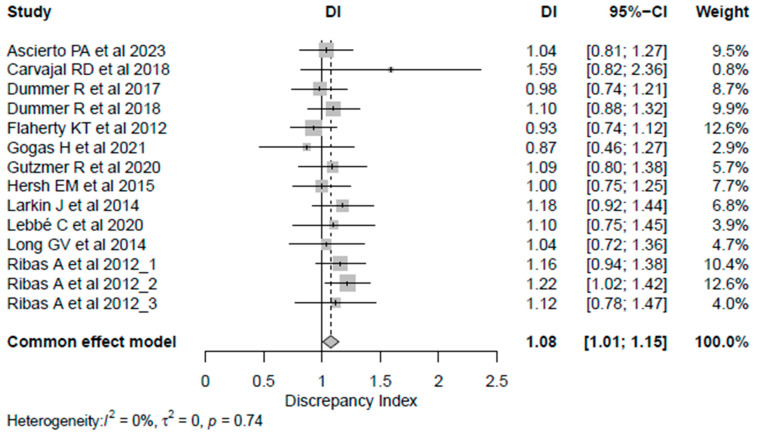
Forest plot for discrepancy index [19,20,21,22,23,24,25,26,27,28,29,30]. Abbreviations: CI: confidence intervals; DI: discrepancy index.

**Figure 2:**
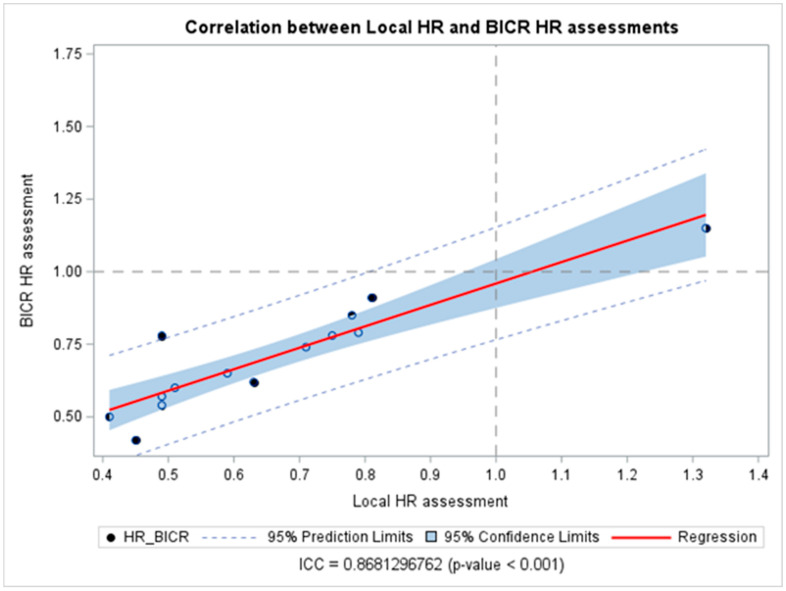
Correlation between hazard ratios obtained from blinded independent central review and local investigators’ assessments. Abbreviations: BICR: blinded independent central review; HR: hazard ratios; ICC: intraclass correlation coefficient.

The DI calculated for each reported PFS is reported in Table 2. Most studies (n = 8, 75%) had a DI > 1, thus indicating that the PFS assessed by BICR tended to be less favorable than the PFS assessed by LIs, while the DI equaled 1 in one study. Despite this, the calculated DI did not show a statistically significant discrepancy between the reported HR_LIs_ and HR_BICR_, except for one HR reported by Ribas A et al. [29] (DI = 1.22, 95% CI 1.02–1.42). Also, all the studies showed the sane significance inference direction, except Carvajal RD et al. [20], which showed a discordance between the two methods of assessment. The overall combined DIs were 1.08 (95% CI 1.01–1.15), indicating an average difference of only 8% between HR_LIs_ and HR_BICR_, confirming a statistically significant difference between the HR obtained by either LIs or BICR; however, the difference was close to 1, indicating a high degree of agreement in the PFS HR estimates overall (Figure 1). When considering cutaneous melanoma trials exclusively, DIs clustered closely around 1, indicating a high degree of concordance between BICR and LI assessments. The results were consistent across univariate analysis for all analyzed subgroups except for double-blinded studies, which showed a significantly higher median inter-quartile range (IQR) DI [1.16 (0.13)] than studies with an open-label masking design [1.0 (0)] (*p* = 0.0076) (Table 3) in the univariate analysis. Also, the meta-regression analysis did not show any potential subgroup difference. The correlation between HRs obtained from LIs and BICR is reported in Figure 2. The ICC was 0.87, *p*-value < 0.001, suggesting a strong correlation between the two assessments. Among the analyzed studies, two showed a relatively larger discrepancy between local and central PFS assessments (Carvajal RD et al. [20] on the upper side, and Flaherty KT et al. [23] on the lower side of Figure 2). Moreover, the Pearson correlation coefficient (*r* = 0.89, 95% CI 0.67–0.96, *p* < 0.0001) in Table S5 in the Supplementary Materials indicates a strong positive correlation between the HR assessed by BICR and LIs. This suggests that as the HR measured by one method increases, the HR measured by the other also tends to increase, and vice versa.

### 3.3. Risk of Bias Assessment Results

**Figure 3:**
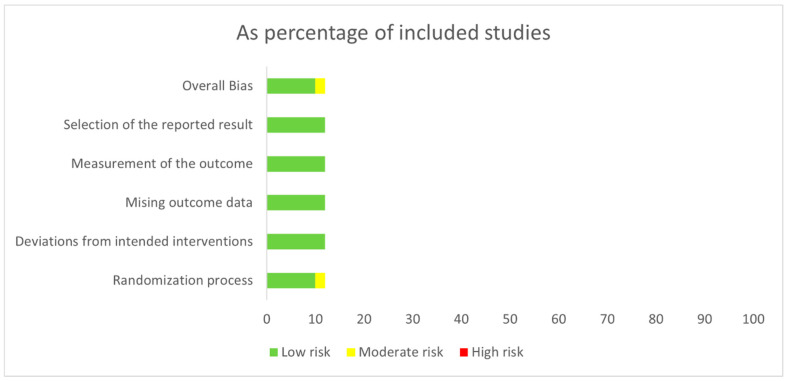
Risk of bias assessments of the studies included in the meta-analysis.

The risk of bias assessment for the studies included in this meta-analysis is presented in Figure 3. In general, ten studies were classified as having a low risk of bias. The remaining two studies by Flaherty KT et al. [23] and Gogas H et al. [24] were found to have a moderate risk of bias, primarily related to the randomization process. This was attributed to their open-label design, which may allow knowledge of the assigned intervention to influence the outcome assessment. A comprehensive evaluation of the risk of bias for each study is provided in Table S6 in the Supplementary Materials.

### 3.4. Publication Bias Assessment

The funnel plot in Figure S2 in the Supplementary Materials illustrates symmetrical appearance, homogeneity, and a minimum sample size variation among the included studies, which indicates the absence of bias except for Carvajal RD et al. [20], which included a smaller sample size than most studies.

## 4. Discussion

Blinded independent central review is widely used as a standard approach in registration trials where PFS is the primary endpoint, as regulatory bodies in the USA and Europe often recommend [31,32]. Our findings do not imply that LI-assessed PFS should universally replace BICR for regulatory purposes; rather, they support a risk-based, trial-specific approach in metastatic cutaneous melanoma, where concordance between LIs and BICR was high and differences rarely changed statistical inference. Despite this, there has been significant debate in the academic community about whether BICR offers a real advantage over assessments conducted by local investigators, especially considering the added costs and operational challenges associated with its use [15]. In this systematic review and meta-analysis, we analyzed data from 12 RCTs comprising 4915 patients with metastatic melanoma. Our findings revealed notable differences between PFS evaluations by LIs and BICR, with LI-based assessments yielding numerically lower hazard ratio estimates compared with BICR. However, the overall magnitude of these differences or biases was relatively minor (8%), with BICR estimating a weaker treatment effect. To contextualize the clinical magnitude of the observed discrepancy, an average 8% difference in HR estimates would be expected to translate into relatively small absolute differences in PFS. For example, in a trial with a median PFS of 10 months in the control arm, an HR of 0.70 based on local investigator assessment would correspond to an estimated median PFS of approximately 14.3 months in the experimental arm, whereas an HR of 0.76 based on BICR would correspond to approximately 13.2 months—an absolute difference of about one month. Such differences are unlikely to be clinically meaningful in most settings and rarely change the overall interpretation of trial outcomes. This result may be explained by the fact that BICR serves as a mechanism to identify and mitigate potential biases that may arise during assessments conducted by LIs. This practice stems from the assumption that LIs, particularly in open-label trials, may inherently anticipate greater efficacy from treatments in the experimental arm compared to those in the control arm [12]. However, in our analysis, these results were primarily driven by a Phase II study testing a novel immunotherapeutic regimen or a double-blinded Phase III study testing a combination of targeted therapy and chemotherapy in uveal melanoma, which showed a higher median DI than other Phase III or open-label trials in cutaneous melanoma. When considering cutaneous melanoma trials exclusively, DIs clustered closely around 1, indicating a high degree of concordance between BICR and LI assessments. These findings challenge the necessity of universally implementing BICR in all RCTs, supporting their appropriate use in select scenarios, primarily Phase II RCTs testing emerging immunotherapeutic approaches. Phase II RCTs are often a critical step in drug development, where important decisions have to be made before investing in the larger, more costly Phase III trials. Therefore, investing in BICR at this stage may warrant serious consideration. The results may also support the value of double-blinded studies as important tools to minimize inherent biases in efficient endpoint assessments, although the Phase III trial in uveal melanoma was complicated by liver-dominant disease, with patients potentially previously exposed to regional therapeutic interventions that may make response assessment more challenging.

It is important to distinguish between systematic bias and methodological variance when interpreting differences between LI and BICR assessments. Systematic bias would imply a consistent directional distortion of treatment effect estimates attributable to investigator behavior or incentives. In contrast, methodological variance reflects structural differences in assessment processes, including censoring rules, confirmation requirements, adjudication procedures, and the timing of radiographic evaluations. Random measurement variability further contributes to dispersion between estimates. In the context of this study, the discrepancy index captures the net effect of these factors and should not be interpreted as direct evidence of investigator bias in the absence of patient-level concordance data.

These results align with those of previous studies [12,13,14]. For instance, an earlier 2024 meta-analysis [13] was conducted by collaborators from Genentech, Inc. in the USA and F. Hoffmann-La Roche Ltd. in Switzerland, collecting HRs for PFS from all Roche-supported cancer clinical trials. The study reported that BICR was more statistically significant and less favorable than LIs (DI = 1.044, 95% CI 1.009–1.081), whereas they had a high agreement as the DI was almost 1. Moreover, they observed that the BICR results did not change the interpretation of the study outcome. On the other hand, these results differ from the most recent meta-analysis by Jacob et al. (2024) [15], covering 24 RCTs up to the end of 2023, which compared PFS evaluations by LIs and BICR in mBC trials. That analysis demonstrated that the BICR assessment yielded numerically lower HR estimates than LIs (DI = 097; 95% CI 0.85–1.10). However, it is essential to highlight the differences in patient populations between our study and the mBC study.

Our meta-analysis revealed a robust agreement and accordance between LI and BICR assessments, affirming the validity of investigator-assessed PFS as a primary endpoint in RCTs involving metastatic melanoma. There are similarities between the magnitude of agreement and accordance between LI and BICR assessments expressed by Pearson’s correlation in this study and those described by Amit et al. (2011) [33] and Lian et al. (2023) [13], or Zhang et al. (2018) [12] and Jacob et al. (2024) [15], who reported a strong positive correlation between both assessments (*r* = 0.94, 95% CI 0.88–0.97 and *r* = 0.95, 95% CI 0.90–0.96, respectively) or (ICC = 0.93, *p* <0.01 and ICC = 0.83, *p* <0.001, respectively).

BICR offers advantages such as mitigating biases, standardizing disease progression or treatment response evaluations across multiple trial sites, minimizing systematic imaging reader biases, and reducing measurement variability, potentially enhancing trial outcomes’ robustness and credibility. In comparison, adopting BICR in clinical trials often introduces significant logistical challenges, including transferring imaging or pathology samples, coordinating data, and facilitating expert reviews. These requirements can result in increased costs and delays, particularly problematic in studies with urgent timelines or constrained resources [11]. Hence, scientists have suggested that BICR should not be universally applied to all RCTs. Instead, its implementation should be guided by a rigorous, case-by-case scientific evaluation, prioritizing trials where the risk of bias is notably high. Such risks are associated with open-label designs, multicenter trials, reliance on subjective endpoints, or extended study durations. In these trials with a higher bias risk, it can serve as a helpful sensitivity check to corroborate local assessments and mitigate bias; however, where there is a low risk of bias, BICR may be unnecessary [11,13,15].

Notably, a higher degree of variability was noted in the KEYNOTE-002 Phase II RCT, which assessed the immunotherapeutic anti-PD-1 monoclonal antibody pembrolizumab [29]. It is well established that conventional RECIST criteria have considerable limitations when applied to immunotherapy for solid tumors, supporting the need for tailored evaluation frameworks such as the Immune-Related Response Evaluation Criteria in Solid Tumors (iRECIST) [34]. These specialized criteria account for the distinctive response patterns associated with immunotherapies, including the phenomenon of pseudo-progression. Pseudo-progression is characterized by an initial apparent increase in tumor burden, manifesting as either an enlargement of target lesions or the appearance of new lesions. This phenomenon may arise from continued tumor growth until a robust antitumor immune response is mounted or due to an increased infiltration of immune cells into the tumor microenvironment, which may falsely suggest tumor progression [35]. A subsequent and often durable response is observed in approximately 10% of cases classified as disease progression under RECIST criteria [35]. Therefore, it is plausible that the discrepancies between LIs and BICR observed in the KEYNOTE-002 trial can be partially attributed to the challenges inherent in accurately assessing progression in the context of immunotherapies.

Our univariate analysis revealed that the median DI was more frequently greater in open-label trials. This suggests that HR_BICR_ assessments were less inclined to favor experimental treatments than HR_LE_ in these settings. The notably high DI values may influence the observed trend reported in two double-blind studies, specifically those conducted by Carvajal et al. (SUMIT) [20] and Ribas et al. (KEYNOTE-002) [29], which also reported statistically inconsistent inferences between two assessments among the included 12 trials, which could be referred to as evaluation variability. Censoring and other unmentioned factors simultaneously played a role in attenuating the treatment effects. As discussed earlier, KEYNOTE-002 [29] was a randomized Phase II trial investigating novel immunotherapeutic agents. On the other hand, the SUMIT trial suggested that the difference in PFS assessments could be explained by liver-dominant disease, with patients potentially previously exposed to regional therapeutic interventions that may make response assessment more challenging. This is in addition to the unique toxicity profile of selumetinib—including visibly apparent adverse effects such as rash, peripheral edema, and elevated creatine phosphokinase—that may have inadvertently influenced site-based assessments of PFS, potentially introducing bias [20]. All immunotherapy trials included in this analysis used RECIST version 1.1, which is known to inadequately capture immune-related response patterns such as pseudo-progression. This limitation may differentially affect BICR and local investigator assessments, as central review applies strict imaging-based confirmation rules without access to evolving clinical context, potentially leading to the earlier classification of progression. The higher variability observed in KEYNOTE-002 [29] likely reflects these methodological constraints rather than assessment bias. It is plausible that the application of immune-adapted criteria such as iRECIST would reduce discordance between assessment strategies; however, no included trial employed iRECIST, precluding direct evaluation.

Our findings indicate the absence of significant systematic bias in this meta-analysis due to the lack of an obvious risk of bias, publication bias, or subgroup analysis, which could support the robustness and generalizability of our results.

This meta-analysis offers several strengths. First, it is the largest analysis to date comparing PFS assessments by LIs and BICR in the context of metastatic melanoma, incorporating data from the latest RCTs. Second, while focusing on a homogeneous patient population, the analysis still considered variations in melanoma subtypes and treatments, with more than 99% of patients having skin melanoma. We conducted additional evaluations to ensure accurate subgroup analysis aligned with the overall results.

This study has several limitations that should be considered when interpreting the findings. First, this was a retrospective, study-level meta-analysis, which precludes assessment of patient-level concordance between BICR and LI assessments, including within-patient discrepancies or inter-reader variability within BICR. Second, only published randomized trials were included, raising the possibility of publication bias, particularly if studies with discordant or null findings were less likely to report both BICR and LI results. Selective reporting within trials cannot be excluded, as some studies provided incomplete statistical information for one assessment method. Third, several unresolved trial-level factors could not be evaluated, including whether patients were monitored post-progression based on local assessments, the protocol-defined interval between local and central review, and the timing of follow-up imaging when discrepancies arose—particularly in cases where treatment continuation was based on investigator judgment. Fourth, the interpretation of masking effects is limited by confounding between study design, melanoma subtype, and treatment class. Double-blind trials in this analysis included heterogeneous therapeutic approaches and the only uveal melanoma study, preventing disentanglement of the independent effects of masking, disease biology, and treatment modality. Fifth, one included trial (KEYNOTE-002) contributed multiple treatment arms, resulting in non-independent discrepancy index estimates. While this may modestly influence precision and heterogeneity metrics, the overall conclusions were consistent when this study was considered descriptively, and findings should be interpreted accordingly. Finally, the study period spanned more than a decade (2012–2023), during which imaging practices and RECIST-based assessment evolved. We were unable to account for heterogeneity in imaging protocols, scanner types, slice thickness, or acquisition parameters across trials. In addition, the limited number of immunotherapy-only trials restricted treatment-class-specific inference.

Finally, we recommend relying on LI-based assessments as the primary evidence while reserving BICR for sensitivity analyses only when scientifically warranted through a study-specific evaluation, which can streamline the delivery of innovative treatments to patients while minimizing costs. Regardless of the decision to implement BICR, we recommend systematic collection and secure storage of radiographic images obtained during the study. This approach facilitates a potential “on-demand” BICR if deemed necessary at a later stage and supports future exploratory analyses. Additionally, the proactive collection of all imaging data may help mitigate potential LI biases, as the possibility of a future BICR could enhance the rigor of local evaluations.

## 5. Conclusions

Our systematic review and meta-analysis of RCTs in metastatic melanoma demonstrated a general alignment between PFS assessments conducted by LIs and BICR. The analysis revealed that the DI, as defined in this study, tended to approach a value of 1, indicating minimal statistically significant systematic bias between local and central evaluations. These findings apply primarily to RCTs in cutaneous melanoma, while results from uveal melanoma should be interpreted cautiously and considered exploratory until additional randomized evidence becomes available. This conclusion is supported by the precisely calculated pooled HR ratios for PFS. Based on these findings, we conclude that while BICR remains an essential tool for minimizing potential biases associated with local assessments, its routine application to all patients in oncological RCTs may not always be warranted. Instead, its use should be carefully tailored to each trial’s specific requirements and risks.

## Figures and Tables

**Table 1:** Characteristics of the included clinical trials.

First Author	Study Name/NCT Identifier	Year of Publication	Sample Size	PFS Follow-Up Time in Months	Study Phase	Masking	Melanoma Subtype	PFS Primary Endpoint	RECIST Version	Investigational Drug(s)	Comparator(s)
Ascierto PA et al. [19]	COLUMBUS	2023	344	60	Phase III	Open-label	Cutaneous	Primary	1.1	Encorafenib + Binimetinib	Encorafenib
Carvajal RD et al. [20]	SUMIT	2018	129	12	Phase III	Double-Blind	Uveal	Primary	1.1	Selumetinib + Dacarbazine	Placebo + Dacarbazine
Dummer R et al. [22]	NEMO	2017	402	18	Phase III	Open-label	Cutaneous	Primary	1.1	Binimetinib	Dacarbazine
Dummer R et al. [21]	COLUMBUS	2018	577	28	Phase III	Open-label	Cutaneous	Primary	1.1	Encorafenib + Binimetinib	Encorafenib or Vemurafenib
Flaherty KT et al. [23]	METRIC	2012	322	8	Phase III	Open-label	Cutaneous	Primary	1.1	Trametinib	Chemotherapy (dacarbazine or paclitaxel)
Gogas H et al. [24]	IMspire170	2021	446	15	Phase III	Open-label	Cutaneous	Primary	1.1	Cobimetinib + Atezolizumab	Pembrolizumab
Gutzmer R et al. [25]	IMspire150	2020	514	30	Phase III	Double-Blind	Cutaneous	Primary	1.1	Atezolizumab + Vemurafenib + Cobimetinib	Placebo + vemurafenib + Cobimetinib
Hersh EM et al. [26]	NCT00864253	2015	529	33	Phase III	Open-label	Cutaneous	Primary	1.0	Nab-paclitaxel	Dacarbazine
Larkin J et al. [27]	coBRIM	2014	495	15	Phase III	Double-Blind	Cutaneous	Primary	1.1	Vemurafenib + Cobimetinib	Vemurafenib + Placebo
Lebbé C et al. [30]	NCT01693068	2020	194	25	Phase II	Open-label	Cutaneous	Primary	1.1	Pimasertib	Dacarbazine
Long GV et al. [28]	COMBI-d	2014	423	18	Phase III	Double-Blind	Cutaneous	Primary	1.1	Dabrafenib + Trametinib	Dabrafenib + Placebo
Ribas A et al. [29]	KEYNOTE-002	2015	540	16	Phase II	Double-Blind	Cutaneous	Primary	1.1	Pembrolizumab	Chemotherapy (paclitaxel + carboplatin, paclitaxel, carboplatin, dacarbazine, or temozolomide)

PFS, progression-free survival; RECIST, Response Evaluation Criteria in Solid Tumors.

**Table 2:** Discrepancy indexes of the studies included in the meta-analysis.

First Author	Study Name	PFS HR (95% CI) Assessed by BICR	*p*-Value	PFS HR (95% CI) Assessed by Local Investigators	*p*-Value	Discrepancy Index (DI)		
						HR (BICR)/ HR (Local)	Lower Limit 95% CI	Upper Limit 95% CI
Ascierto PA et al. [19]	COLUMBUS	0.74 (0.6–0.92)	0.003	0.71 (0.58–0.87)	0.0005	1.04	0.81	1.27
Carvajal RD et al. [20]	SUMIT	0.78 (0.48–1.27)	0.32 *	0.49 (0.28–0.84)	0.01 *	1.59	0.82	2.36
Dummer R et al. [22]	NEMO	0.62 (0.47–0.8)	<0.001	0.63 (0.48–0.82)	<0.001	0.98	0.74	1.21
Dummer R et al. [21]	COLUMBUS	0.54 (0.41–0.71)	0.0001	0.49 (0.37–0.64)	<0.0001	1.10	0.88	1.32
Flaherty KT et al. [23]	METRIC	0.42 (0.29–0.59)	<0.001	0.45 (0.33–0.63)	<0.001	0.93	0.74	1.12
Gogas H et al. [24]	IMspire170	1.15 (0.88–1.5)	0.3	1.32 (1.02–1.71)	N/A	0.87	0.46	1.27
Gutzmer R et al. [25]	IMspire150	0.85 (0.67–1.07)	0.16 *	0.78 (0.63–0.97)	0.02 *	1.09	0.80	1.38
Hersh EM et al. [26]	NCT00864253	0.79 (0.63–0.99)	0.04	0.79 (0.63–0.99)	0.04	1.0	0.75	1.25
Larkin J et al. [27]	coBRIM	0.6 (0.45–0.79)	0.0003	0.51 (0.39–0.68)	<0.001	1.18	0.92	1.44
Lebbé C et al. [30]	NCT01693068	0.65 (0.45–0.94)	0.019	0.59 (0.42–0.83)	0.002	1.10	0.75	1.45
Long GV et al. [28]	COMBI-d	0.78 (0.59–1.04)	N/A	0.75 (0.57–0.99)	0.03	1.04	0.72	1.36
Ribas A et al. [29]	KEYNOTE-002	0.57 (0.45–0.73)	<0.0001	0.49 (0.38–0.62)	<0.0001	1.16	0.94	1.38
Ribas A et al. [29]	KEYNOTE-002	0.5 (0.39–0.64)	<0.0001	0.41 (0.32–0.52)	<0.0001	1.22	1.02	1.42
Ribas A et al. [29]	KEYNOTE-002	0.91 (0.71–1.16)	N/A	0.81 (0.63–1.05)	0.12	1.12	0.78	1.47

* statistically inconsistent inferences. Abbreviations: BICR, blinded independent central review; CI, confidence interval; HR, hazard ratio; N/A, not available; PFS, progression-free survival.

**Table 3:** Comparison of the discrepancy index between different study groups.

Characteristics	Type	Number of Studies	DI Median (IQR)	*p*-Value
Study phase	Phase II	4	1.14 (0.08)	0.0519
	Phase III	10	1.04 (0.12)	
Masking	Double-blind	7	1.16 (0.13)	0.0076 *
	Open-label	7	1.0 (0.17)	
RECIST version	RECIST_v1.0	1	1.0 (0)	0.2284
	RECIST_v1.1	13	1.10 (0.12)	
Melanoma type	Cutaneous	13	1.09 (0.12)	0.0683
	Uveal	1	1.59 (0)	
Randomization ratio	1:1	9	1.10 (0.12	0.2778
	2:1	3	0.98 (0.17)	
	3:1	2	1.32 (0.55)	
Sponsor	AstraZeneca	1	1.59 (0)	0.2709
	Celgene Corporation	1	1.0 (0)	
	EMD Serono	1	1.10 (0)	
	F. Hoffmann–La Roche/Genentech	3	1.09 (0.30)	
	GlaxoSmithKline	2	0.99 (0.11)	
	Merck & Co	3	1.16 (0.09)	
	Novartis and Array BioPharma	1	1.10 (0)	
	Pfizer	2	1.01 (0.06)	
Hazard ratio significance level_LI	Significant	12	1.10 (0.15)	0.2408
	Not significant	1	1.12 (0)	
	Not available	1	0.87 (0)	
Hazard ratio significance level_ BICR	Significant	9	1.10 (0.16)	0.9968
	Not significant	2	1.09 (0.72)	
	Not available	3	1.08 (0.08)	
PFS follow-up time	≤17 months	7	1.04 (0.29)	0.1797
	>17 months	7	1.16 (0.10)	

* *p*-value < 0.05. Abbreviations: BICR, blinded independent central review; DI, discrepancy index; IQR, inter-quartile range; LI, local investigator; PFS, progression-free survival; RECIST, Response Evaluation Criteria in Solid Tumors.

## Data Availability

The data used to generate this study’s findings are publicly available, and the codes used to analyze them are available upon request from the corresponding author.

## Supplementary Materials

The following supporting information can be downloaded at: https://www.mdpi.com/article/10.3390/cancers18040710/s1, Table S1 shows the PRISMA checklist [36]. Tables S2–S4 contain the search strategy in different databases. Figure S1 demonstrates the PRISMA flowchart that shows the study selection process. Table S5 explains the agreement assessment of PFS between BICR and LIs. Figure S2 presents the funnel plot for publication bias assessment. Table S6 presents the risk of bias assessments of the studies included in the meta-analysis.
