# Supplementary material for: Survival Assessment by Central Review vs. Local Investigator in Metastatic Melanoma: A Systematic Review and Meta-Analysis
Source: Cancers (Basel). 2026 Feb 22;18(4):710. doi: 10.3390/cancers18040710 (PMC12938985; doi:10.3390/cancers18040710)
Supplement: Supplementary file 1 [file cancers-18-00710-s001.zip › Systematic Review and Meta-Analysis Supplementary Materials.23Dec25.IE.pdf]

Supplemental Materials

Survival Assessment by Independent Central Review Versus Local Investigator in Metastatic Melanoma: A Systematic Review and Meta-Analysis.

Authors and Affiliations

Islam Eljilany[1]; Eissa Jafari[2,3]; Abdullah Alhumaid[3,4]; Zeynep Eroglu[1]; Andrew S Brohl[1]; Lilit Karapetyan[1]; Joseph Markowitz[1]; Nikhil I Khushalani[1]; Patrick Hwu[1] and Ahmad A. Tarhini[1,5]

[1]Department of Cutaneous Oncology, H. Lee Moffitt Cancer Center and Research Institute, Tampa, FL 33612, USA.

[2]Department of Pharmacy Practice, College of Pharmacy, Jazan University, Jazan, Saudi Arabia.

[3]Department of Pharmacotherapy and Translational Research and Center for Pharmacogenomics and Precision Medicine, College of Pharmacy, University of Florida, Gainesville 32610, Florida.

[4]Department of Clinical Pharmacy, College of Pharmacy, University of Hail, Hail, Saudi Arabia.

[5]Department of Immunology, H. Lee Moffitt Cancer Center and Research Institute, Tampa, FL 33612, USA.

***Corresponding Author:**

Ahmad A. Tarhini, M.D., Ph.D.

Departments of Cutaneous Oncology and Immunology

H. Lee Moffitt Cancer Center and Research Institute

10920 McKinley Dr. Tampa, Florida 33612, United States

Phone: 813-745-8581

Email: Ahmad.Tarhini@moffitt.org

Table S1. PRISMA checklist

PRISMA 2020 Checklist - Systematic reviews and meta-analyses

Section and Topic	Item #	Checklist item	Reported on page #
**TITLE**			
Title	1	Identify the report as a systematic review.	1
**ABSTRACT**			
Abstract	2	See the PRISMA 2020 for Abstracts checklist.	4
**INTRODUCTION**			
Rationale	3	Describe the rationale for the review in the context of existing knowledge.	6
Objectives	4	Provide an explicit statement of the objective(s) or question(s) the review addresses.	7
**METHODS**			
Eligibility criteria	5	Specify the inclusion and exclusion criteria for the review and how studies were grouped for the syntheses.	8
Information sources	6	Specify all databases, registers, websites, organisations, reference lists and other sources searched or consulted to identify studies. Specify the date when each source was last searched or consulted.	7-8 Supplementary Table S2-S4
Search strategy	7	Present the full search strategies for all databases, registers and websites, including any filters and limits used.	7-8
Selection process	8	Specify the methods used to decide whether a study met the inclusion criteria of the review, including how many reviewers screened each record and each report retrieved, whether they worked independently, and if applicable, details of automation tools used in the process.	8
Data collection process	9	Specify the methods used to collect data from reports, including how many reviewers collected data from each report, whether they worked independently, any processes for obtaining or confirming data from study investigators, and if applicable, details of automation tools used in the process.	8
Data items	10a	List and define all outcomes for which data were sought. Specify whether all results that were compatible with each outcome domain in each study were sought (e.g. for all measures, time points,	8

		analyses), and if not, the methods used to decide which results to collect.	
	10b	List and define all other variables for which data were sought (e.g. participant and intervention characteristics, funding sources). Describe any assumptions made about any missing or unclear information.	9
Study risk of bias assessment	11	Specify the methods used to assess risk of bias in the included studies, including details of the tool(s) used, how many reviewers assessed each study and whether they worked independently, and if applicable, details of automation tools used in the process.	8-9
Effect measures	12	Specify for each outcome the effect measure(s) (e.g. risk ratio, mean difference) used in the synthesis or presentation of results.	9
Synthesis methods	13a	Describe the processes used to decide which studies were eligible for each synthesis (e.g. tabulating the study intervention characteristics and comparing against the planned groups for each synthesis (item #5)).	8
	13b	Describe any methods required to prepare the data for presentation or synthesis, such as handling of missing summary statistics, or data conversions.	9
	13c	Describe any methods used to tabulate or visually display results of individual studies and syntheses.	9
	13d	Describe any methods used to synthesize results and provide a rationale for the choice(s). If meta-analysis was performed, describe the model(s), method(s) to identify the presence and extent of statistical heterogeneity, and software package(s) used.	9
	13e	Describe any methods used to explore possible causes of heterogeneity among study results (e.g. subgroup analysis, meta-regression).	9
	13f	Describe any sensitivity analyses conducted to assess robustness of the synthesized results.	9
Reporting bias assessment	14	Describe any methods used to assess risk of bias due to missing results in a synthesis (arising from reporting biases).	9
Certainty assessment	15	Describe any methods used to assess certainty (or confidence) in the body of evidence for an outcome.	9
**RESULTS**			
Study selection	16a	Describe the results of the search and selection process, from the number of records identified in the search to the number of studies included in the review, ideally using a flow diagram.	10

	16b	Cite studies that might appear to meet the inclusion criteria, but which were excluded, and explain why they were excluded.	10<br>Supplementary Figure S1
Study characteristics	17	Cite each included study and present its characteristics.	10<br>Table 1
Risk of bias in studies	18	Present assessments of risk of bias for each included study.	13<br>Supplementary Table S6 and Figure 3
Results of individual studies	19	For all outcomes, present, for each study: (a) summary statistics for each group (where appropriate) and (b) an effect estimate and its precision (e.g. confidence/credible interval), ideally using structured tables or plots.	13-19<br>Table 1-3<br>Figures 1-3
Results of syntheses	20a	For each synthesis, briefly summarise the characteristics and risk of bias among contributing studies.	13<br>Supplementary Table S6 and Figure 3
	20b	Present results of all statistical syntheses conducted. If meta-analysis was done, present for each the summary estimate and its precision (e.g. confidence/credible interval) and measures of statistical heterogeneity. If comparing groups, describe the direction of the effect.	13<br>Figure 1
	20c	Present results of all investigations of possible causes of heterogeneity among study results.	13<br>Figures 1-2, Supplementary Figure 3, Table S3
	20d	Present results of all sensitivity analyses conducted to assess the robustness of the synthesized results.	13<br>Figures 1-2, Supplementary Figure 3, Table S3

Reporting biases	21	Present assessments of risk of bias due to missing results (arising from reporting biases) for each synthesis assessed.	13 Supplementary Table S6 and Figure 3
Certainty of evidence	22	Present assessments of certainty (or confidence) in the body of evidence for each outcome assessed.	13
**DISCUSSION**			
Discussion	23a	Provide a general interpretation of the results in the context of other evidence.	20
	23b	Discuss any limitations of the evidence included in the review.	23
	23c	Discuss any limitations of the review processes used.	23
	23d	Discuss implications of the results for practice, policy, and future research.	23-24
**OTHER INFORMATION**			
Registration and protocol	24a	Provide registration information for the review, including register name and registration number, or state that the review was not registered.	7
	24b	Indicate where the review protocol can be accessed, or state that a protocol was not prepared.	Not Prepared
	24c	Describe and explain any amendments to information provided at registration or in the protocol.	Not applicable
Support	25	Describe sources of financial or non-financial support for the review, and the role of the funders or sponsors in the review.	24
Competing interests	26	Declare any competing interests of review authors.	2
Availability of data, code and other materials	27	Report which of the following are publicly available and where they can be found: template data collection forms; data extracted from included studies; data used for all analyses; analytic code; any other materials used in the review.	Not applicable

From: Page MJ, McKenzie JE, Bossuyt PM, Boutron I, Hoffmann TC, Mulrow CD, et al. The PRISMA 2020 statement: an updated guideline for reporting systematic reviews. BMJ 2021;372:n71. doi: 10.1136/bmj.n71
For more information, visit: http://www.prisma-statement.org/

Table S2. Search Strategy_PubMed

	Search string	Number of studies found
#1	("melanoma"[MeSH terms] OR "melanoma"[Title/abstract] OR "cutaneous cancer"[Title/abstract] OR "skin cancer"[Title/abstract] OR "melanocytic"[Title/abstract])	181,051
#2	("progression free survival"[MeSH Terms] OR "progression free survival"[All Fields] OR "progression-free survival"[All Fields] OR "survival progression-free"[All Fields] OR "survival progression free"[All Fields] OR "PFS"[All Fields] OR "time to progress"[All Fields] OR "TTP"[All Fields] OR "progression free rate"[All Fields] OR "progression-free rate"[All Fields] OR"progression free interval"[All Fields] OR "progression-free interval"[All Fields] OR "PFI"[All Fields])	97,709
#3	("randomized controlled trial"[Publication Type] OR "randomized controlled trial"[Title/abstract] OR "randomised controlled trial"[Title/abstract] OR "randomized clinical trial"[Title/abstract] OR "randomised clinical trial"[Title/abstract] OR "clinical trial"[Title/abstract] OR "clinical study"[Title/abstract] OR "controlled clinical trial"[Title/abstract] OR "randomized"[Title/abstract] OR "RCT"[Title/abstract])	1,166,390
#4	("Phase III"[Title/abstract] OR "Phase 3"[Title/abstract] OR "Phase II"[Title/abstract] OR "Phase 2"[Title/abstract] OR "Phase IIa"[Title/abstract] OR "Phase IIb"[Title/abstract] OR "Phase 2a"[Title/abstract] OR "Phase 2b"[Title/abstract] OR "phase-2"[Title/abstract] OR "phase-II"[Title/abstract] OR "phase-2a"[Title/abstract] OR "phase-2b"[Title/abstract] OR "phase-IIa"[Title/abstract] OR "phase-IIb"[Title/abstract] OR "phase-3"[Title/abstract] OR "phase-III"[Title/abstract])	147,363
#5	#1 AND #2 AND #3 AND #4	300
#6	English only	298

Table S3. Search Strategy_Cochrane

	**Search string**	Number of studies found
#1	(melanoma:ti,ab,kw OR "cutaneous cancer":ti,ab,kw OR "skin cancer":ti,ab,kw OR melanocytic:ti,ab,kw)	8008
#2	("progression free survival":ti,ab,kw OR "progression-free survival":ti,ab,kw OR "survival progression-free":ti,ab,kw OR "survival progression free":ti,ab,kw OR pfs:ti,ab,kw OR "time to progress":ti,ab,kw OR ttp:ti,ab,kw OR "progression free rate":ti,ab,kw OR "progression-free rate":ti,ab,kw OR "progression free interval":ti,ab,kw OR "progression-free interval":ti,ab,kw OR pfi:ti,ab,kw)	41982
#3	("randomized controlled trial":pt OR "randomized controlled trial":ti,ab,kw OR "randomised controlled trial":ti,ab,kw OR "randomized clinical trial":ti,ab,kw OR "randomised clinical trial":ti,ab,kw OR "clinical trial":ti,ab,kw OR "clinical study":ti,ab,kw OR "controlled clinical trial":ti,ab,kw OR randomized:ti,ab,kw OR rct:ti,ab,kw)	1303080
#4	("phase III":ti,ab,kw OR "phase 3":ti,ab,kw OR "phase II":ti,ab,kw OR "phase 2":ti,ab,kw OR "phase IIa":ti,ab,kw OR "phase IIb":ti,ab,kw OR "phase 2a":ti,ab,kw OR "phase 2b":ti,ab,kw OR "phase-2":ti,ab,kw OR "phase-II":ti,ab,kw OR "phase-2a":ti,ab,kw OR "phase-2b":ti,ab,kw OR "phase-IIa":ti,ab,kw OR "phase-IIb":ti,ab,kw OR "phase-3":ti,ab,kw OR "phase-III":ti,ab,kw)	148957
#5	#1 AND #2 AND #3 AND #4	825
#6	English only	824

Table S4. Search Strategy_Embase

	**Search string**	Number of studies found
#1	('melanoma'/exp OR 'melanoma':ti,ab OR 'cutaneous cancer':ti,ab OR 'skin cancer':ti,ab OR 'melanocytic':ti,ab)	283,759
#2	('progression free survival'/exp OR 'progression free survival':ab,ti OR 'progression-free survival':ab,ti OR 'survival progression-free':ab,ti OR 'survival progression free':ab,ti OR 'pfs':ab,ti OR 'time to progress':ab,ti OR 'ttp':ab,ti OR 'progression free rate':ab,ti OR 'progression-free rate':ab,ti OR 'progression free interval':ab,ti OR 'progression-free interval':ab,ti OR 'pfi':ab,ti)	248,631
#3	('randomized controlled trial'/exp OR 'randomized controlled trial':ti,ab OR 'randomised controlled trial':ti,ab OR 'randomized clinical trial':ti,ab OR 'randomised clinical trial':ti,ab OR 'clinical trial':ti,ab OR 'clinical study':ti,ab OR 'controlled clinical trial':ti,ab OR 'randomized':ti,ab OR 'rct':ti,ab)	1,609,308
#4	('phase III':ti,ab OR 'phase 3':ti,ab OR 'phase II':ti,ab OR 'phase 2':ti,ab OR 'phase IIa':ti,ab OR 'phase IIb':ti,ab OR 'phase 2a':ti,ab OR 'phase 2b':ti,ab OR 'phase-2':ti,ab OR 'phase-II':ti,ab OR 'phase-2a':ti,ab OR 'phase-2b':ti,ab OR 'phase-IIa':ti,ab OR 'phase-IIb':ti,ab OR 'phase-3':ti,ab OR 'phase-III':ti,ab)	291,257
#5	#1 AND #2 AND #3 AND #4	1092
#6	English only	1087

Figure S1. PRISMA Flowchart showing the study selection process

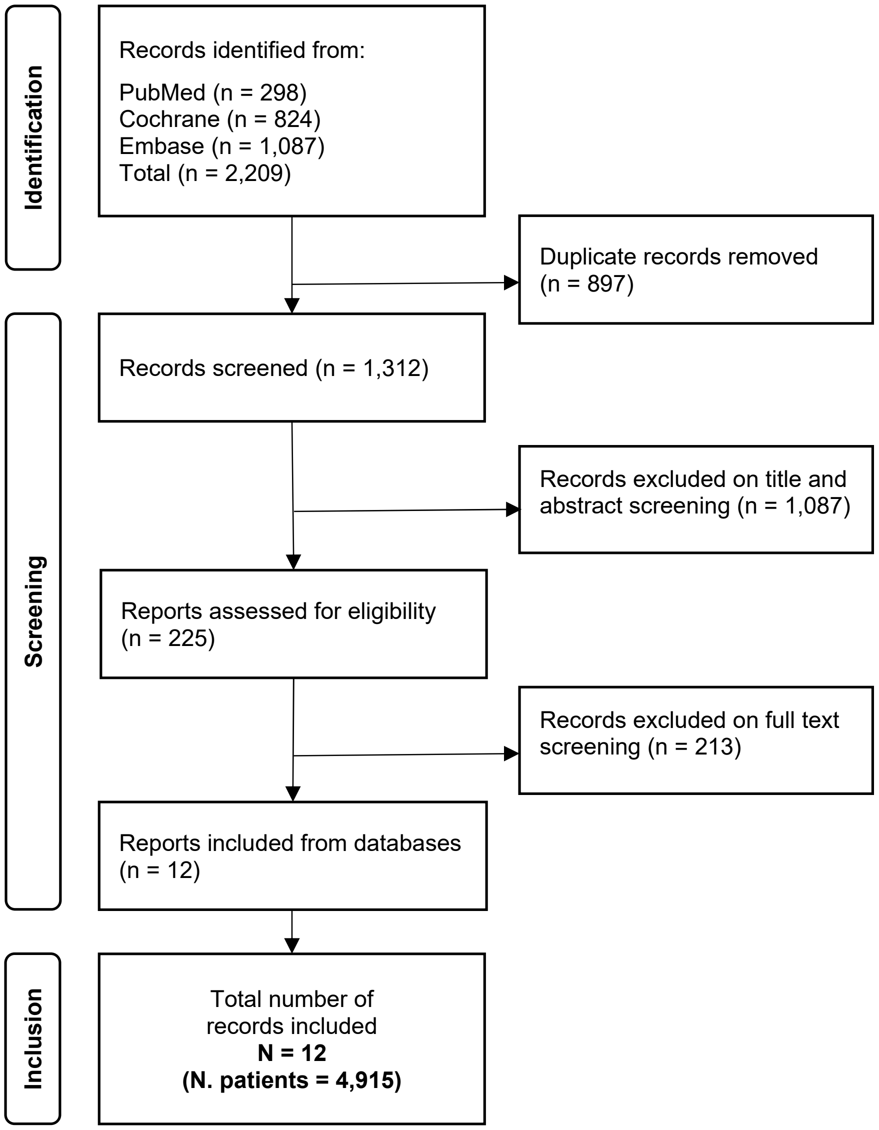

Table S5. Agreement assessment of PFS between BICR and LI

**Number of comparisons**	***r* (95% CI)**	**P-value**
Overall 14	0.89 (0.67-0.96)	<0.0001
r: Pearson's correlation coefficient between log (HR BICR) and log (HR LI)		

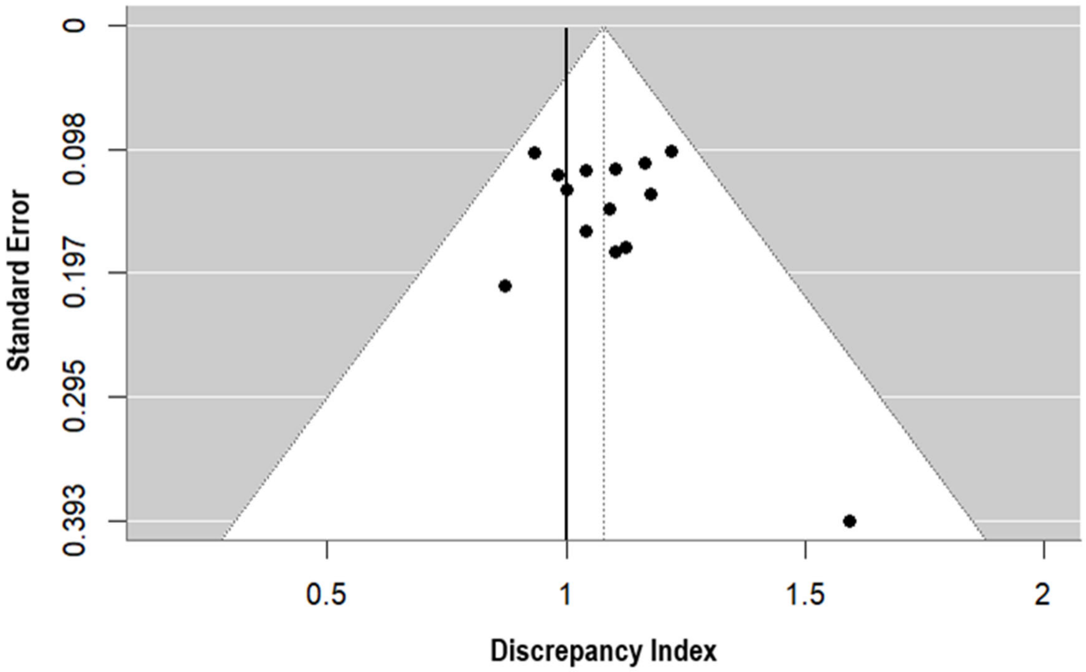

Figure S2. Funnel plot for publication bias assessment

The dotted vertical line represents the calculated discrepancy index (1.08) however, the solid vertical line represents the normal discrepancy index.

Table S6. Risk of bias assessments of the studies included in the meta-analysis

Study ID	Weight	Randomization process	Deviations from intended interventions	Missing outcome data	Measurement of the outcome	Selection of the reported result	Overall Bias
Ascierto PA et al_2023	1	Low	Low	Low	Low	Low	Low
Carvajal RD et al_2018	1	Low	Low	Low	Low	Low	Low
Dummer R et al_2017	1	Low	Low	Low	Low	Low	Low
Dummer R et al_2018	1	Low	Low	Low	Low	Low	Low
Flaherty KT et al_2012	1	Moderate	Low	Low	Low	Low	Moderate
Gogas H et al_2021	1	Moderate	Low	Low	Low	Low	Moderate
Gutzmer R et al_2020	1	Low	Low	Low	Low	Low	Low
Hersh EM et al_2015	1	Low	Low	Low	Low	Low	Low
Larkin J et al_2014	1	Low	Low	Low	Low	Low	Low
Lebbé C et al_2020	1	Low	Low	Low	Low	Low	Low
Long GV et al_2014	1	Low	Low	Low	Low	Low	Low
Ribas A et al_2012	1	Low	Low	Low	Low	Low	Low
